# Responses to River Inundation Pressures Control Prey Selection of Riparian Beetles

**DOI:** 10.1371/journal.pone.0061866

**Published:** 2013-04-17

**Authors:** Matt J. O'Callaghan, David M. Hannah, Ian Boomer, Mike Williams, Jon P. Sadler

**Affiliations:** 1 School of Geography, Earth and Environmental Sciences, The University of Birmingham, Birmingham, United Kingdom; 2 Environment Agency, Exeter, United Kingdom; Duke University, United States of America

## Abstract

**Background:**

Riparian habitats are subjected to frequent inundation (flooding) and are characterised by food webs that exhibit variability in aquatic/terrestrial subsidies across the ecotone. The strength of this subsidy in active riparian floodplains is thought to underpin local biodiversity. Terrestrial invertebrates dominate the fauna, exhibiting traits that allow exploitation of variable aquatic subsidies while reducing inundation pressures, leading to inter-species micro-spatial positioning. The effect these strategies have on prey selection is not known. This study hypothesised that plasticity in prey choice from either aquatic or terrestrial sources is an important trait linked to inundation tolerance and avoidance.

**Method/Principal Findings:**

We used hydrological, isotopic and habitat analyses to investigate the diet of riparian Coleoptera in relation to inundation risk and relative spatial positioning in the floodplain. The study examined patch scale and longitudinal changes in utilisation of the aquatic subsidy according to species traits. Prey sourced from terrestrial or emerging/stranded aquatic invertebrates varied in relation to traits for inundation avoidance or tolerance strategies. Traits that favoured rapid dispersal corresponded with highest proportions of aquatic prey, with behavioural traits further predicting uptake. Less able dispersers showed minimal use of aquatic subsidy and switched to a terrestrial diet under moderate inundation pressures. All trait groups showed a seasonal shift in diet towards terrestrial prey in the early spring. Prey selection became exaggerated towards aquatic prey in downstream samples.

**Conclusions/Significance:**

Our results suggest that partitioning of resources and habitat creates overlapping niches that increase the processing of external subsidies in riparian habitats. By demonstrating functional complexity, this work advances understanding of floodplain ecosystem processes and highlights the importance of hydrological variability. With an increasing interest in reconnecting rivers to their floodplains, these invertebrates represent a key functional element in ensuring that such reconnections have demonstrable ecological value.

## Introduction

Riverine landscapes and their associated floodplains are dynamic environments characterised by high levels of physical habitat heterogeneity and turnover [1]. Longitudinal and lateral structuring of these habitats is controlled fundamentally by the river flow regime [2] and geomorphology, notably sediment supply [3]. Levels of connectivity between the channel and wider landscape are variable [4] with often strong flows of nutrients and food resources [5], [6], [7], [8]. Floodplains are vulnerable to direct and indirect anthropogenic disturbance, becoming increasingly degraded by pressures of urbanisation, pollution, dam impoundment, water abstraction [9] and climate change [10]. As a result, more dynamic channel planforms (such as wandering channels and braided floodplain systems) have declined dramatically to a point where they are regarded as one of the world's most endangered types of freshwater systems [11]. The complex interconnectedness of in-channel, riparian and floodplain zones has been demonstrated hydrologically, geomorphologically [12], biogeochemically [13] and ecologically [4], [14], [15], [16]. This ecological dynamic is partially responsible for the conservation significance of floodplains [17], [18], [19], [20], [21] with flooding (inundation) as a structuring force for the communities [22], leading to clear functional variability in life forms and traits, especially in the numerically abundant invertebrate fauna [23]. Concurrently, floodplains contribute significant ecosystem services, not least their role in mitigating against flooding impacts [24].

Coleoptera associated with riparian margins are numerically dominant and highly adapted invertebrates [22], [25]. The apparent similarity of the species found in riparian coleopteran communities has been hypothesised as a rare example of a lack of ‘intrageneric isolation’ [26], that is multiple species occupying the same niche within individual microhabitats, indicating the utilisation of mechanisms to reduce competitive interactions. Common to all riparian specialists are traits (behavioural and/or morphological) which are beneficial under the environmental pressures of inundation, potentially high sediment temperatures and low moisture. In more stochastic environments, strong trait-based responses may be required [27], [28], with species being ‘filtered out’ [29] from continued habitat and associated resource use if they lack the necessary traits. Characteristic coleopteran traits in this habitat include high reflectivity [30], flattened bodies [31], avoidance behaviour [32], spatial positioning [33], [34] and seasonal changes in habitat choice [35], [36], [37]. The last three of these mechanisms enable species to tolerate the high levels of inundation-driven disturbance [22], [38]. Where there is a strong seasonal element of flooding the inundation pressure may be reduced by timing of lifecycles or translocation from the habitat. For instance in the UK, highest flows are typically observed during the winter, when adults move inland and to higher ground [39]. However, year-round, episodic high flows, e.g. associated with summer convective rainfall [40] are also possible, subjecting adult communities active in riparian habitats to strong selective pressures. Specific traits reducing flood inundation pressures serve to spatially delineate community composition along longitudinal and lateral gradients [22]; changes to the flooding frequency or magnitude can further alter this composition [38], and cause short to medium term variability in abundance and assemblage composition [41], [42]. When traits are matched to local habitat and environmental conditions, the match allows dominance and maximises resource use [43]. The high level of riparian habitat reworking excludes many other species, allowing species with specific traits to fully utilise available prey. However, the presence of multiple traits within an assemblage [23] and microhabitats existing within the matrix of local habitat [19], [44], also suggests multiple strategies for minimising inundation pressures. Although these traits are utilised within individual disturbance events, this environment is characterised by frequent and stochastic disturbance, which is likely to reinforce their value but also the functional consequences of their possession. We seek to address a critical research gap in this article by testing the hypothesis that the traits that enable species to inhabit disturbed floodplains also drive prey choice under differing environmental conditions.

Understanding how complex assemblages utilise the resource and react to environmental pressures is essential for understanding the ecological functioning of floodplains. The extent of the aquatic subsidy to predatory Coleoptera is known to vary longitudinally, rising from 40% in headwater streams to 80% in higher order, lowland rivers [45], [46], which is at least partly due to increased downstream productivity as well as prey availability. Other invertebrate studies of the riparian zone have used stable isotope analysis (SIA) to examine the strength of aquatic subsidies to Aranea [47], [48], Orthoptera [49], Formicidae and Coleoptera [46], although these have not distinguished between the functional traits of the species present in this important ecotone environment. That said SIA techniques provide an efficient and increasingly well understood mechanism for investigating prey sourcing [50], and with invertebrates, analysis of whole organisms is useful for providing a baseline ‘average’ reflecting long term patterns of consumption [51]. The level of uptake of aquatic resources by riparian consumers has been observed to vary spatially and seasonally between taxa [45], [47], [52]. Predatory invertebrates with a lifecycle at least partially dependent on bare and exposed sediments situated in the active floodplain, are likely to have a stronger dependence on aquatic prey items than itinerant species that utilise short-term influxes. There is some evidence that specialist predator abundances are linked to emergence levels of aquatic insects [52], although it is unclear whether this abundance is enabled by the subsidy, or whether the two groups have a level of life-cycle synchronicity, predator emergence coinciding with maximum prey abundances. Within-species spatial variation in subsidy level [42], [53] indicates that dietary plasticity is an important strategy for riparian predators, a concept however, which remains untested. As temporal hydrologic variability decreases downstream from variable headwaters [54], we hypothesise that the ‘value’ of different traits will vary, favouring different functional groups and altering the stakes of the risk : subsidy trade off for riparian consumers.

Although there is an increasing amount of work on riparian invertebrate community dynamics there is limited knowledge about their functional response to hydrological (and habitat) variability and how functional groupings change under different inundation conditions [23], [41]. In the light of on-going anthropogenic impacts, global threats to floodplain integrity and changing hydrological regimes envisaged under present climate change scenarios [10], the ecological function of this important group needs to be better understood.

We aimed to investigate functional processes in riparian Coleoptera, using stable isotope analysis to identify environmentally, spatially and temporally driven variation in dietary composition occurring amongst functional groups. We achieved this through addressing a series of three linked objectives to: (i) define hypothetical functional groups, using dispersal related traits, (ii) examine variations in dietary composition between these groups along a lateral gradient away from the river's edge and longitudinally downstream, and over three seasons (iii) investigate the role of different inundation pressures on prey choice by the different functional groups.

We achieved our aims and objectives and address in the Discussion the contributions the study has made to floodplain ecology research. We also highlight some of the methodological issues with the work in relation to the temporal and spatial scales of the study and the role of detritivores and phytophagous species in nutrient processing.

## Results

### Invertebrate data and functional groups

The samples were derived from 1,695 terrestrial Coleoptera, 973 potential aquatic prey and 260 potential terrestrial prey. Some samples (Collembola and aphids) comprised multiple individuals (3–5) due to the small size of organisms. Isotopic values were obtained for 50 terrestrial prey samples, 262 aquatic prey samples (reduced to 130; see methods) and 366 predatory terrestrial coleopteran samples. Consumers were assigned to 5 functional groups defined by statistical analysis of morphological variation and behavioural characteristics (Table 1).

**Table 1:** Functional groups of predatory terrestrial Coleoptera sampled from ERS on the upper River Severn, giving example member species, geographical and micro-spatial preferences, and morphological characteristics.

Functional group	Micro-spatial preference	Morphology	Example member species
*Group 1* Specialist ground beetles	HeadwatersMobile within patch	Long legs & wings	*B. atrocaeruleum* *B. tibiale*
*Group 2* Specialist ground beetles	LowlandWetted edge	Long legs & wings	*B. punctulatum* *B. decorum*
*Group 3* Low affinity ground beetles	Damp ground	Long legs	*B. tetracolum* *P. albipes*
*Group 4* Ground beetles with no ERS association	In land	Long legs	*P. madidus*
*Group 5* Specialist non-ground beetles	HeadwatersRaised ERS	Shorter legs & wings	*Stenus* spp.*C. 5-punctata*

Measured morphological variation analysed via ANOVA showed significant difference between the leg : body length ratios of all ground beetles, specialist click and rove beetles (p<0.001: F. 82.04, df 2 and 75), all ground beetles had significantly longer legs. Between wing: body length ratio of specialist ground beetles and all other beetles (including species of non-specialist ground beetles) also differed significantly (p<0.001: F 102.62, df; 2 and 75)), the specialist ground beetles had longer wings. Generalised Linear Modelling further refined these groups. The specialist ground beetles were subdivided, into a distinct headwater grouping, including *Bembidion atrocaeruleum* (Stephens, 1828) and *Bembidion decorum* (Zanker in Panzer, 1800) (AIC 82.61, p<0.005: d 8.17, 19df) and a lowland associated grouping, including *Bembidion punctulatum* (Drapaiz, 1821) and *Bembidion tibiale* (Duftschmid, 1812) (AIC 77.77, p <0.05: d 27.99, df 19). Specialist click and rove beetles which lack both the longer legs and wings of ground beetles also exhibited a high affinity with headwater habitat (AIC 93.49, p<0.005: d 21.07, df 19). The resulting five groups, defined by morphological and modelling of distribution, comprised; headwater specialist ground beetles (group 1), lowland specialist ground beetles (group 2), low affinity ground beetles (group 3), no affinity ground beetles (group 4) and specialist non-ground beetles (group 5).

### Environmental and Habitat Variation

**Figure 1:**
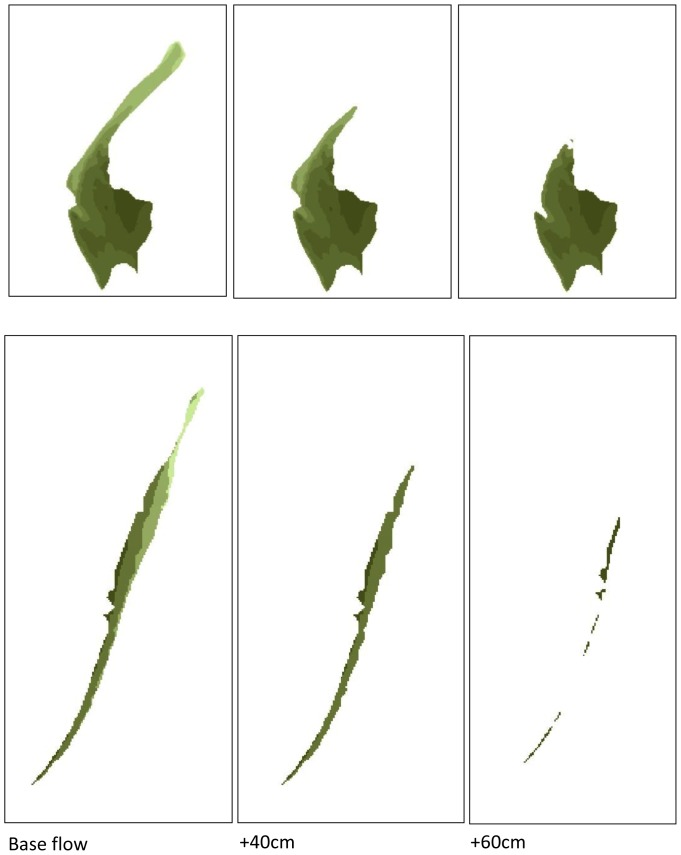
Digital Elevation Models showing the different extent of habitat loss under low, medium and high flows for representative gravel bars with (a) elevated profile and (b) shallow profile. Figure 1a shows patch 10, a large area, complex habitat patch, of which only 28% is submerged when levels are 1 m above base flows; Figure 1b shows patch 15, a low elevation habitat patch, of which 100% is submerged under the same conditions.

**Figure 2:**
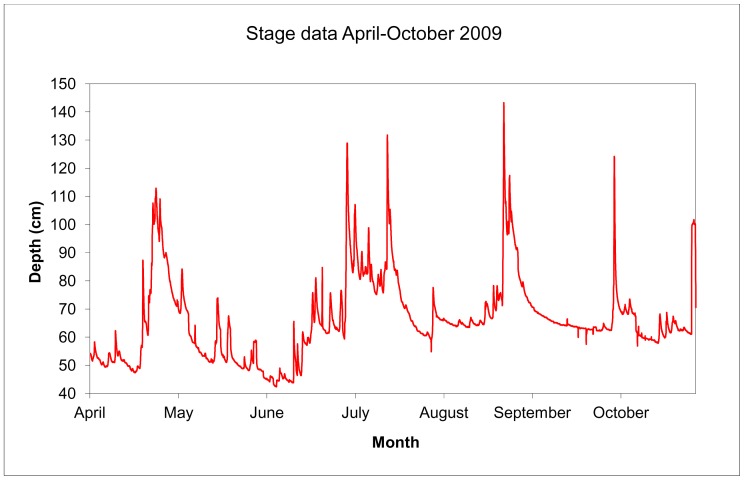
Daily depth readings for the River Severn at Llandinam Gravels between April-October 2009 showing variations around the baseline depth of 54 cm on April 4^th^, the date on which gravel bars were surveyed.

Digital elevation models (DEM), river level and flow (discharge) data were used to identify three inundation classes for analysis of patch scale processes (Table 2; and methods for details). Five bars experienced low inundation pressure (<50% loss of habitat), 6 bars experienced moderate pressure (51–90% loss) and 9 bars experienced high pressure (>90%), examples of inundation extent are shown in Figure 1. River depth (level) was higher consistently during autumn and winter associated with higher rainfall. The spring-summer maximum depth of 143.3 cm was exceeded seven times between October and March, the peak event being 176.2 cm, which inundated all patches (1.23 m above the depth measured in April 2009 when the d-GPS surveys were conducted). Figure 2 shows the daily river depth during the period of peak invertebrate activity in the study (April–October 2009), six bars experienced total inundation during this period, whilst the five least affected bars lost less than 50% of available area under the highest flows in September 2009 (1.4 m above April 2009). The depth data also shows that the duration of inundation events varied between bars, from several weeks in July for shallow profile bars, to hours for steeper profile bars in short-lived pulse events in July, September and October. The extent, or presence of habitat availability, was compromised for prolonged periods on the lower bars, requiring greater use of refugia by resident fauna; more elevated bars retain the shingle habitat under all but autumn-winter flows.

**Table 2:** Inundation classes of studied habitat patches (bars), with percentage habitat lost with a 1 m increase above base flow measurements (summer maxima), or for patches lower than 1 m, at the point at which they were submerged.

Patch	% of habitat submerged with 1 metre increase over base flow	Inundation susceptiblity
1	100	High
2	86	Moderate
3	89	Moderate
4	58	Moderate
5	53	Moderate
6	100	High
7	100	High
8	100	High
9	100	High
10	28	Low
11	51	Moderate
12	39	Low
13	13	Low
14	39	Low
15	100	High
16	96	High
17	93	High
18	40	Low
19	92	High
20	62	Moderate

Correlation analysis of environmental and inundation variables conducted to establish covariance that might influence invertebrate behaviour indicated the presence of significant relationships between inundation and extent of vegetation cover (negative), also bar area and length of wetted edge (positive) (Table 3). An assessment was then made which of the correlating variables had the strongest environmental effect and these were selected for exploration in isotopic modelling; inundation and bar area being selected.

**Table 3:** Spearman's rank correlation coefficients for environmental variables, showing significant relationships between the area and edge length of patch, area of patch and incline, and frequency of inundation and incline.

	Heterogeneity	Vegetation	Incline	Area	Substrate Phi	Edge length	Inundation
Heterogeneity	1.000						
Vegetation	0.398	1.000					
Incline	0.218	0.265	1.000				
Area	−.083	0.158	**0.612** *	1.000			
Substrate Phi	−0.356	−0.084	0.263	−0.042	1.000		
Edge length	0.086	0.110	0.38	**0.703** *	0.14	1.000	
Inundation	−.145	−0.357	−**0.544** *	0.286	0.835	0.515	1.000

* significant at>0.05.

### Isotope data

**Figure 3:**
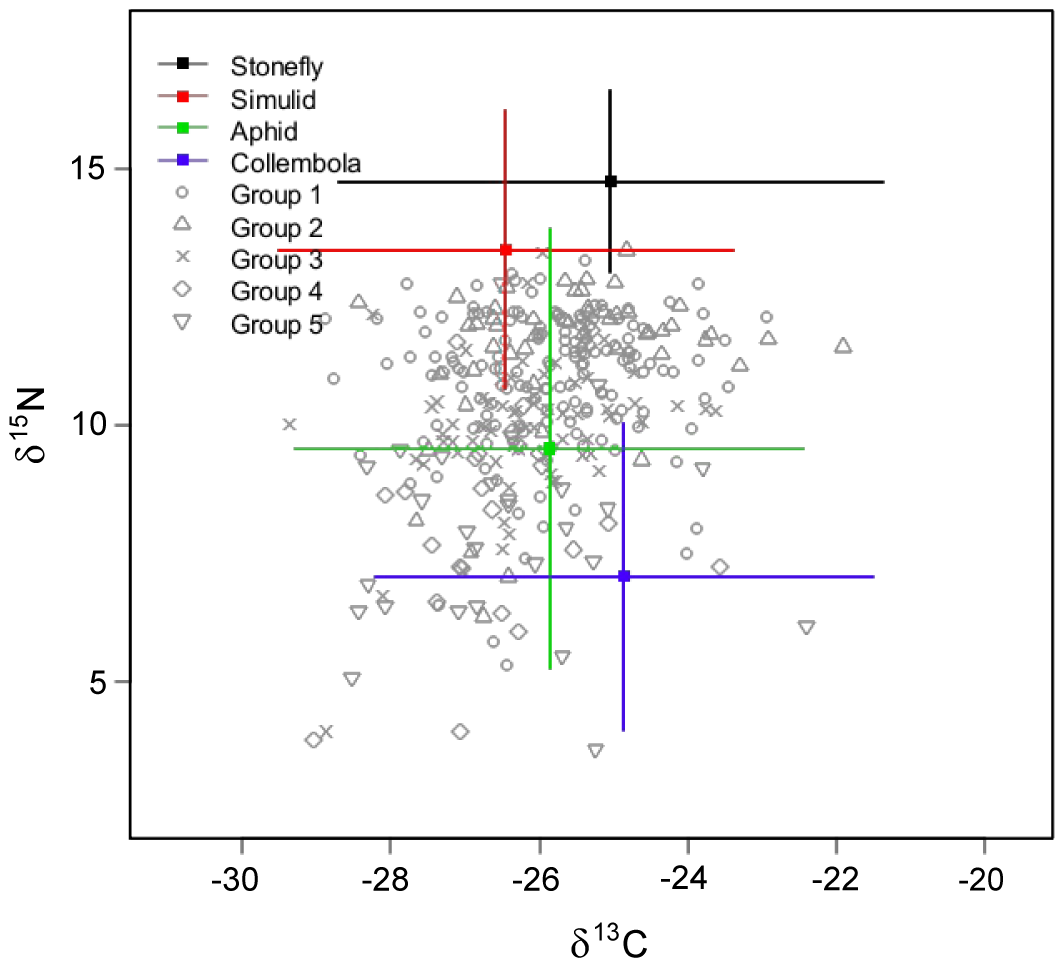
Biplot of principle identified prey sources and consumer data. Aquatic invertebrates (blackflies and stoneflies show greatest δ^15^N, relative to terrestrial invertebrates (springtails and aphids). The majority of consumer data lies within observed prey values, indicative of dietary contributions from both aquatic and terrestrial prey. Mean isotopic values for prey items are shown ± SD, individual consumer values are shown.

Exploration of the potential prey within the SIAR (Stable Isotopes in R) [55] mixing model indicated that four invertebrate groups formed the majority of all prey selected: simuliids, Plecoptera, Collembola and aphids. Simuliid larvae showed greater abundance in comparison to similarly sized Chironomidae, whilst Plecoptera typically emerge directly onto the riparian zone, rather than from the river surface, or from vegetation (e.g. caddis and mayflies). These potential prey exhibited a clear separation of isotopic values, with aquatic sources (simuliids and Plecoptera) relatively enriched in δ^15^N compared to terrestrial sources (Collembola and aphids), with values between 4.07–12.63 δ^15^N for the former and 1.44–8.26 δ^15^N for the latter. Coleopteran values consistently lay between those of terrestrial and aquatic sources, indicating contributions from both prey groups (Figure 3).

### Isotopic niche positioning

**Figure 4:**
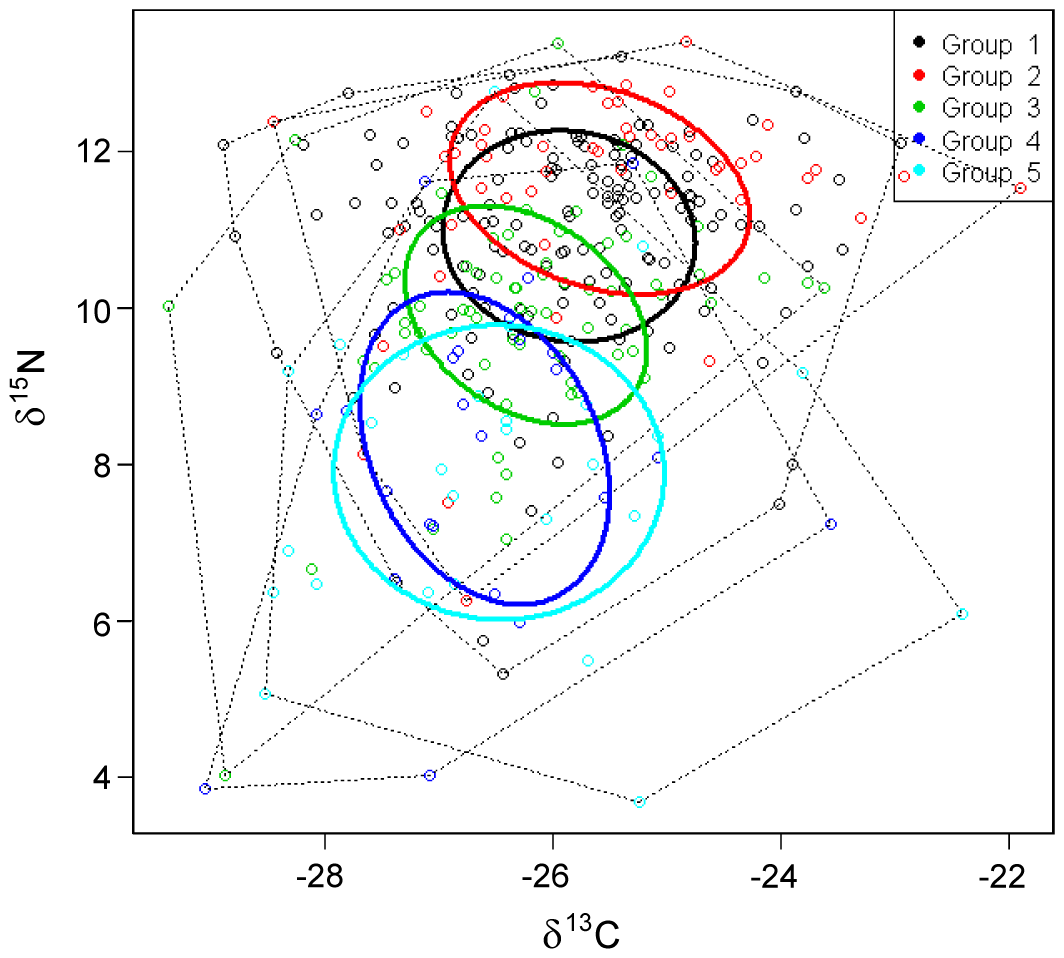
The isotopic niche areas for hypothesized functional groups (1 is headwater specialist ground beetles, 2 is lowland specialist ground beetles, 3,weak affinity ground beetles, 4, ground beetles with no habitat affinity; 5, specialist non-ground beetles associated with headwaters). Dotted lines indicate the convex hull for each group, the extent of all individuals' plotted isotopic values; ellipses represent the probable area in which the population's plotted values are likely to be found. Greater levels of δ^15^N are indicative of greater contributions from aquatic prey items.

Estimation of isotopic niche area for member species from predefined coleopteran functional groups indicates differing levels of aquatic prey utilisation (Figure 4). Non- specialist ground beetles (Group 4) and *Stenus* spp. and *Coccinella 5-punctata* (Linnaeus, 1758) (Group 5) and showed low levels of δ^15^N enrichment, indicative of a terrestrially sourced diet. Conversely, two specialist ground beetles with different preferred positions, stream edge and whole patch (*B. atrocaeruleum* and *B. punctulatum* respectively) exhibited the highest levels of δ^15^N enrichment, indicating greater use of aquatic prey. Overlapping the basal and top positions a weak specialist, *Bembidion tetracolum* (Say, 1823) exhibited median levels of δ^15^N enrichment.

### Physical habitat variability

Exploration of influence of habitat variables in SIAR identified two controls of prey choice, but only for *B. tetracolum* which has a weak affinity to the floodplain habitat. In coarser substrates (Phi class −5 to −6) the terrestrial component of diet increased from 50% to 72%. Terrestrial prey subsidy ranged from 50–70% as the levels of habitat heterogeneity on the bars increased. Bar area, which was highlighted as a potential influence in the environmental correlations, showed no influence on prey selection in any group.

### Effect of lateral sampling position

**Figure 5:**
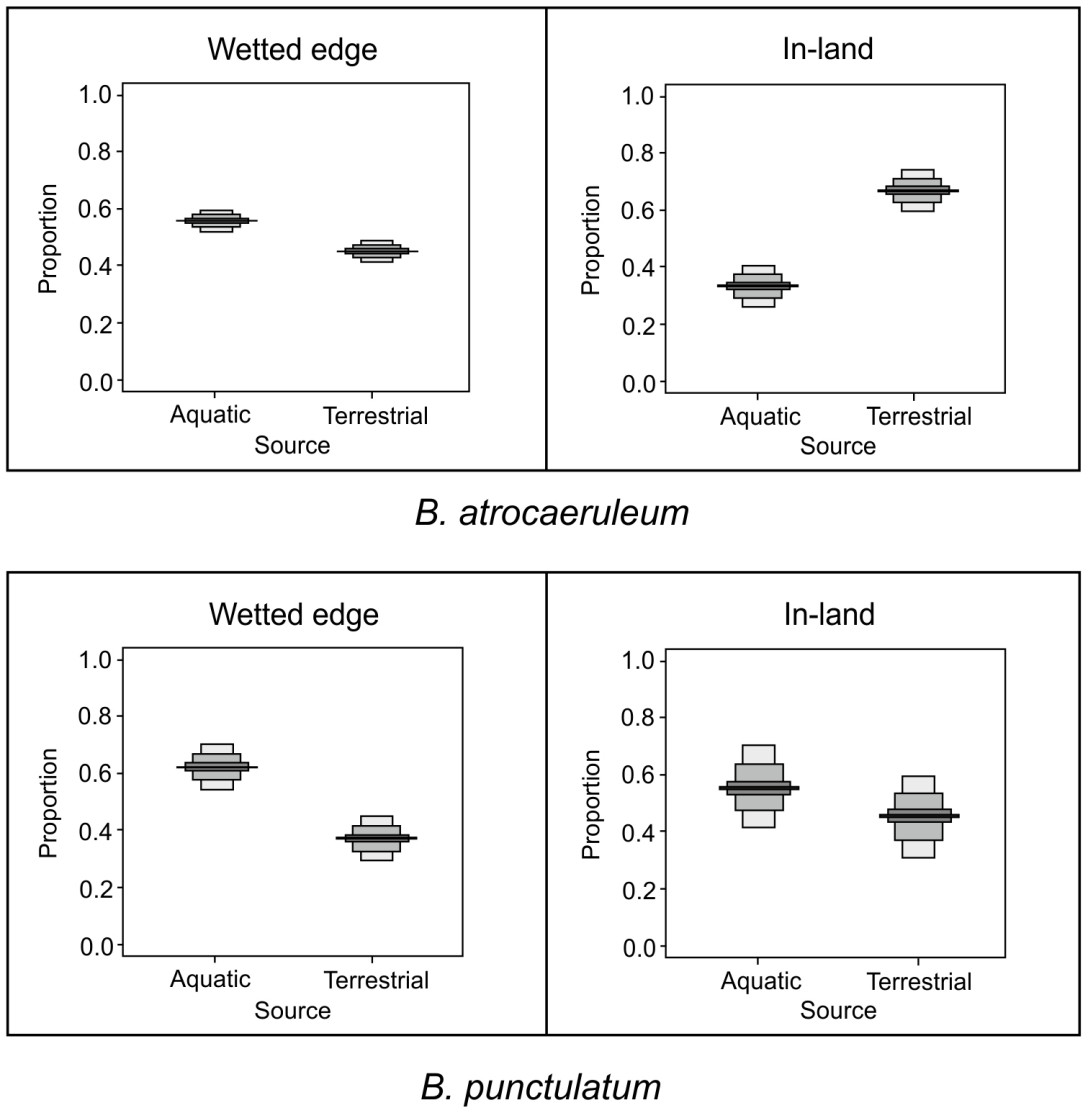
Probability density function of dietary proportions of two species of specialist ground beetles, *B. atrocaeruleum* (a) and *B. punctulatum* (b) illustrating the relative dietary contributions made by aquatic and terrestrial prey according to whether samples were collected from the wetted edge of the habitat patch, or inland, at the point of permanent vegetation. The mid-line represents their mean with 25%, 75% and 95% credibility intervals.

Different dietary composition was detected for all groups according to the sampling distance from the stream edge. *B. atrocaeruleum* (Group 1), known to be mobile within the habitat and associated with headwaters, showed the strongest variation in diet (Figure 5a). Median (most probable) values revealed 60% aquatic and 40% terrestrial contributions at the wetted edge, compared to a 30%∶70% split further inland. *B. punctulatum* (Group 2), known to have a preference for the wetted edge area of the disturbed riparian habitat, showed a similar but smaller decrease in aquatic contributions inland from 62% to 55% (Figure 5b). *Stenus* spp. and *C. 5-punctata* (Group 5) and non-specialised ground beetles (Group 4) showed no change according to sampling position, at 70% terrestrial prey for ground beetles with no affinity and >95% terrestrial for specialist non-ground beetles. *B. tetracolum* (Group 3) showed a stable dietary composition, at 30% aquatic derived prey, regardless of sampling position.

### Seasonal variation

**Figure 6:**
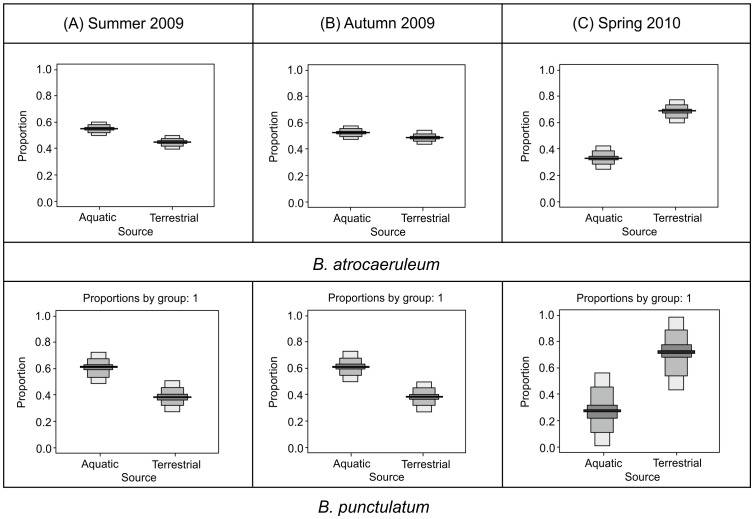
Probability density function of seasonal change in dietary composition in *B. atrocaeruleum* and *B. punctulatum* showing summer (A) 2009, autumn (B) 2009 and spring (C) 2010. The mid-line represents the mean with 25%, 75% and 95% credible intervals shown.

Specialist riparian ground beetles exhibited a strong seasonal variation in dietary composition, with the importance of aquatic prey declining sharply in spring samples (Figure 6). *B. atrocaeruleum* exhibited 50% aquatic prey, 50% terrestrial prey in summer and autumn, changing to 32% aquatic, 68% terrestrial in the spring. *B. punctulatum* exhibited consistent 60∶40% aquatic: terrestrial split for summer/autumn changing to 35%∶65% in the spring.

### Inundation and resource acquisition

**Figure 7:**
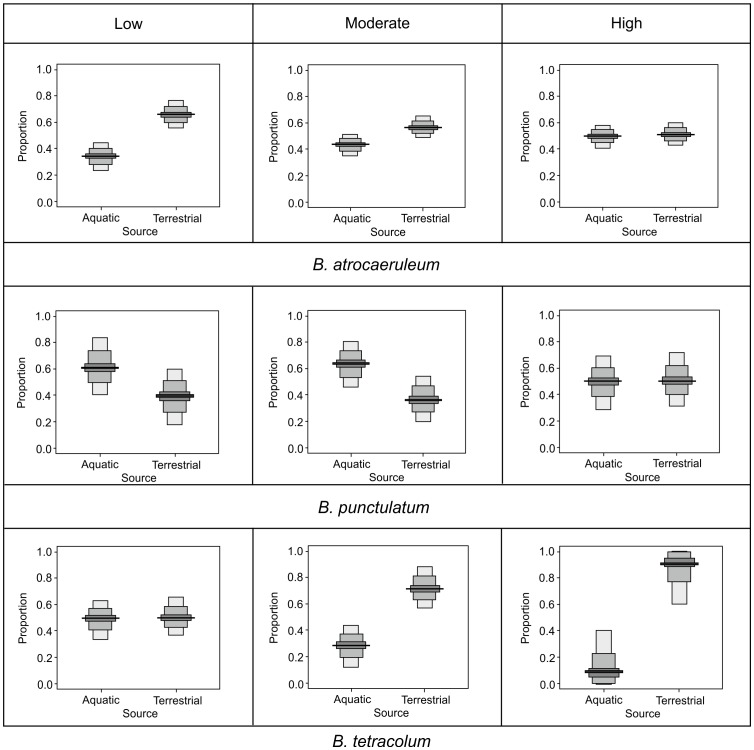
Probability density functions of ground beetle species from each of the groups with a level of association with the disturbed floodplain habitat, (A) *B. atrocaeruleum*, (B) *B. punctulatum* and (C) *B. tetracolum*, showing variation in dietary composition grouped by inundation levels (Low, Moderate, High; see Table 1 for descriptions of levels). The mid-line represents the mean with 25%, 75% and 95% credible intervals shown.

The three numerically dominant species B. *atrocaeruleum* (headwater), *B. punctulatum* (lowland) and *B. tetracolum* (low habitat affinity) are all from the same genus, and are similar sizes (∼5 mm). Median values of dietary proportions indicated differing responses to inundation pressures (Figure 7). *B. atrocaeruleum* and *B. punctulatum* show values indicative of their preferred micro-spatial positioning, which converge under high inundation levels, as available habitat is reduced and encounters with alternative prey increase, stream-edge *B. punctulatum* reduces its intake of aquatic prey under higher levels of inundation pressure, whilst the mobile *B. atrocaeruleum* reduces its intake of terrestrial prey under the same conditions. *B. tetracolum* appears to switch rapidly to a terrestrially based diet under moderate inundation risk, which may be indicative of flood aversion behaviour. On bars with low inundation pressures, *B atrocaeruleum* showed values of 35% aquatic and 65% terrestrial dietary composition, which changed to 42% aquatic and 58% terrestrial under moderate inundation pressures and 45% aquatic, 55% terrestrial under high inundation pressures. Under low to moderate pressures, the values of *B. punctulatum* show a dominance of aquatic subsidy (60%), declining to 35% under high pressure. *B. tetracolum* has both aquatic and terrestrial sources at ∼50% under low pressure, with the aquatic subsidy declining to 30% at moderate levels and 15% under high pressure.

**Figure 8:**
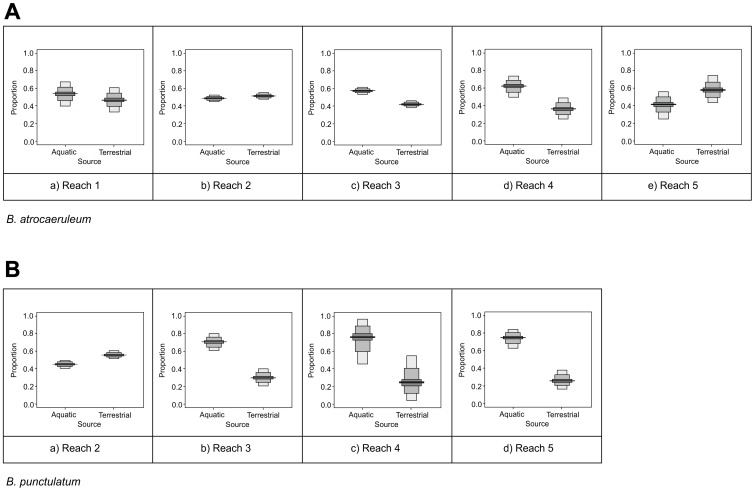
Probability density functions of longitudinal variation in prey source for the two specialist species, *B. atrocaeruleum* (a) and *B. punctulatum* (b), along a headwater to lowland floodplain gradient. The mid-line represent their median and the shaded boxes representing the 50%, 75% and 95% credible intervals from dark to light grey.

The longitudinal patterns of variation across the additional 15 sites revealed strong trends downstream, especially for *B. punctulatum* (Figure 8a), which had a 55% aquatic signal from samples taken in the headwaters to a maximum of 80% at the site 170 km downstream. Conversely, *B. atrocaeruleum* (Figure 8b) maintained a terrestrially dominated diet (70%) from the headwaters to the most downstream sampling location (60%), albeit with an increase in aquatic subsidy for mid-catchment sampling points. Finally, *B. tetracolum* exhibited a switch from 65% terrestrial diet at its highest sampling point to a consistent 55% aquatic diet at the two sampling areas furthest downstream.

## Discussion

The results demonstrate the presence of strong variations in the choice of prey by riparian Coleoptera across multiple gradients. The evidence indicates that these choices are in part driven by behavioural and morphological traits that determine the resilience of representative species to inundation pressures. Dietary composition shows that under the highest levels of disturbance (autumn-winter flooding), all species employ avoidance strategies until inundation pressure becomes reduced in spring. These data also suggest that the beetles do not undergo total quiescence during the winter and maintain at least some level of activity away from the active floodplain. Finally, our results show evidence of exaggerated relative source contributions with increasing distance from the headwaters, with the species which preferentially inhabit the stream edge markedly increasing uptake of aquatic prey at downstream sites. We discuss each element in turn.

### Trait possession and influence on prey selection

Trait groupings were defined by behavioural and morphological characteristics [56], and these groupings became clearly functionally delineated when relative isotopic niche positions were investigated. An increasing utilization of aquatic subsidies was present when the species possessed traits that reduced the risks associated with high flows. Previous investigations have tended to class functionality by taxa; ant raiding parties [57] web building spiders [58], predatory beetles [20], [21], [59], but there has been little or no success in establishing how species with life-cycles tied to the floodplain may differ functionally from generalist, opportunistic species. Our evidence confirms, to our knowledge, for the first time that not only do riparian invertebrates make different prey selection choices (as observed by [45], [52]) but, that there is a gradation of trait-driven specialisms, which dictate functional responses to the high-flow events characteristic of the habitat. This supports recent research on desert riparian arthropods, which suggests concurrent low flow drivers[16]. This flow-related relationship demonstrates the persistent influence of the stream into terrestrial environments, continuing the in-stream, trait-driven responses that have elsewhere been demonstrated [27], [60]. Species may possess a total affinity to the habitat (e.g. *C. 5-punctata*), but lack the traits which allow full utilisation of the subsidies available. Conversely, a combination of beneficial traits (e.g. mobility, positioning preference) which provide advantages during disturbance [30] allows flexible, and therefore broader utilisation of available subsidies. *B. tetracolum* is known to exhibit morphological plasticity, with wing length increasing with proximity to rivers [25], therefore the individuals in this study may be assumed to be within the upper range of wing size for this species, with the pressures of flooding selecting strengthened macroptery. In contrast, other species with stronger, or total affinity to the habitat, are more strongly aligned with the riparian habitat and do not exhibit downstream assemblage heterogeneity or utilize other riparian habitats. Whilst micro-spatial positioning has been demonstrated [33], [34], [44] as evidence of resource partitioning amongst specialist invertebrates, we believe that this is the first that trait-driven resource partitioning has been shown to extend to prey selection in these riparian systems.

### Influence of habitat variability on prey selection

Although micro-spatial positioning of species is believed to be controlled by various physical components of the landscape, including sediment calibre, vegetation levels, and humidity (e.g. [44]), the only species where any of these induced a prey selection response is *B. tetracolum*, which has a low affinity to the habitat. Its response to sediment calibre showed a reduction in aquatic prey on larger substrates, and highest levels of aquatic prey at the lowest level of habitat heterogeneity. Both of these variables are tied inherently to inundation: coarser sediments with greater inundation [38] and increased heterogeneity symptomatic of terrestrialisation of the riparian habitat [20] and reduced permeability for aquatic prey [61]. The level of hydrological variation is the primary driver of habitat formation/removal in floodplains [3]. It is possible that the observed responses of *B. tetracolum* to these variables are an indirect measure of the role of changing flows, higher inundation which results in coarser calibre sediments, also reduces access to aquatic prey, and low heterogeneity provides greater permeability for emergent insects, increasing access to aquatic prey.

### Lateral influence of aquatic prey subsidies

Variation in the strength of aquatic influence on the isotopic signal of consumers with differing traits illustrates strong functional differences with the riparian coleopteran fauna. As the biomass of emerging and stranded aquatic invertebrates drops off rapidly within a few metres of stream edge [47], species which are highly dependent on the subsidy must necessarily place themselves at great ‘risk’ of inundation by staying close to their prey. The other alternative is to employ dietary plasticity, so that under adverse conditions, alternative prey are selected. Some species do exhibit a strong preference for stream edge positioning (e.g. *B. punctulatum, B. decorum*) and use greater proportion of aquatic prey. Similar species with equally high dispersal potential (e.g. *B. atrocaeruleum*) exhibit different behaviour, with individuals typically showing greater within patch mobility [34]. Whilst the majority of the individuals of the *B. punctulatum/B. decorum* will be found close to the stream edge, *B. atrocaeruleum* is less densely clustered. The former strategy allows for a greater, more reliable uptake of the aquatic subsidy but potentially places an entire local population at risk from inundation events; the latter strategy reduces access to the aquatic subsidy, but in the event of flooding, a larger proportion of the local population avoids the disturbance. When we tested whether these positioning choices influenced prey preference, all of the *Bembidion* species in this study (regardless of grouping) demonstrated levels of dietary plasticity between stream edge individuals and those sampled further inland, with increasing levels of terrestrial subsidy at inland sampling points. Given the relative impermeability of the riparian zone to the aquatic subsidy, this increase in prey sourcing is to be expected, as terrestrial items become more abundant than aquatic, but it also supports the hypothesis that prey-switching is an important trait in these species, allowing them to make best use of available resources.

### Seasonal variation in prey choice

The strength of this capacity for dietary plasticity is best demonstrated by data on seasonal variations in isotopic signals of consumers. This seasonal element has been observed before [46], [62], although this was within the context of shifting levels of subsidy tied to emergence rates from the river. Our study, based on data collected over 12 months, appears to substantiate the behavioural observations made of European and FennoScandian riparian communities [35], [39], where the default overwintering strategy is to move inland, away from the active channel and thereby removing the population from higher winter flows with potential to rework the floodplain habitat. We hypothesised that as this movement begins in early autumn, it might be possible to detect an obligative shift in diet by riparian consumers, driven both by reduced prey and habitat availability. Our findings indicate that this is the case for all functional groups, even for those with the stream-edge preference. In addition, the strength of this switch toward terrestrial indicates, we suggest, that the overwintering sites are not characterized by total quiescence, but levels of activity that allow enough prey consumption as to alter the isotopic signal of the community. This appears to be the first time that such a shift has been demonstrated in species usually described as having total affinity to the disturbed riparian habitat.

### Inundation pressure as a driver of prey selection

By analysing a geographically proximate population, where environmental variables rather than phenotypic variation are most likely to drive observed variation between bars, we could first test that inundation is the demonstrable factor influencing prey selection (after eliminating habitat characteristics associated with within-patch distributions). Tellingly, those species with traits less beneficial under the disturbance regime were absent from highly inundated patches, as such our data covers only the groups with strong locomotive and/or flight abilities, which were all species of *Bembidion*. At low levels of inundation pressure, there is evidence of resource partitioning between the two species with strongest avoidance traits, with the stream edge species dominated by aquatic and the mobile species by terrestrial isotopic signals. The convergence of these dietary contributions under heightened inundation pressures is indicative of reduced foraging area. As water levels rise, stream edge species migrate up the floodplain [11], encountering more terrestrial prey; whilst mobile species have a greater likelihood of entering the stream edge zone and encountering aquatic prey items. Both responses indicate an opportunistic plasticity in diet that is only mildly affected by flooding pressures. Their mutual dispersal abilities allow them to persist within the habitat (rather than emigrating) and exploit its resources with reduced risk of mortality. The observed, extreme change in prey selection by *B. tetracolum* is indicative of its lack of specialism. *B. tetracolum* is able to opportunistically take aquatic prey items under low risk conditions, but forced by a relative lack of useful traits to abandon the habitat and its subsidy under higher inundation conditions. Species-specific variations in population recovery have been found following major flood events [22], [42]; our data seems to indicate that alongside flood survival mechanisms, continued ability to utilise resources may play a part in these species-specific variations.

### Downstream changes in prey selection

The increasing contribution of aquatic prey to *B. punctulatum* downstream is in accord with studies of higher order rivers [42], [46], but the trend is less strong in *B. atrocaeruleum* and *B. tetracolum*. Elsewhere, we mention that *B. atrocaeruleum* is associated with headwater habitats [63], although it persists for considerable distances downstream (>150 km). The within-patch mobility is appropriate for habitat vulnerable to the unpredictable high flow events characteristic of high altitude streams. It ensures that a proportion of the local population has reduced exposure to sudden rises in flow. However, there is a trade off, as it also reduces the local population's total access to aquatic subsidies. Habitat further downstream has a less flashy hydrological response and greater area of floodplain. Consequently stream-edge positioning incurs less sudden inundation risk. Under these conditions, traits which favour stream-edge positioning have optimum value, as the whole local population can benefit from the increased stability to utilise the subsidy. The exclusion of *B. atrocaeruleum* may indicate a reduction in the efficiency of its traits under lowland, downstream conditions, the temporary rise in subsidies perhaps indicative of a convergence of trait value at mid-points in the river.

### Conclusions and significance

Although easily overlooked due to their physical size and the presence of more charismatic species (e.g. birds), the invertebrate fauna of floodplains represent a major component of floodplain biodiversity. This study demonstrates that hydrologically driven pressures of the stream:riparian ecotone require the possession of specific traits. Without these traits, species are either unable to process the aquatic subsidy, enhancing its movement onto the floodplain, or may only do so under low flow conditions.

Subtle changes in behaviour and the strength of physical traits dictate the optimum positioning of different beetle species, altering their functional contribution to the riparian zone. High affinity species, with relatively weak dispersal traits, have reduced access to the potential subsidy available from the adjacent stream due to their positioning above the zone at greatest risk of flooding. However, this positions them to utilise available terrestrial prey, suggesting that they possess traits that fit them for the specific demands of this micro-habitat. Strong dispersal traits better fit species to utilise the aquatic subsidy, but a second trait filter acts upon able dispersers that favours different strategies under the different hydrological regimes occurring downstream. We have demonstrated that long-term patterns of local hydrology will determine the baseline isotopic signal of predatory Coleoptera. Beyond the scope of this study, and an area that seems ripe for further exploration, is the importance of individual events to this fauna. Utilising tissues (wings or reproductive organs) with rapid isotopic turnover rates may provide a mechanism to examine these short-term processes, eliminating the influence of chitinous material which although has some isotopic turnover [64] may retain a strong larval isotopic signature [65].

Observed abundances of riparian Coleoptera in floodplain habitats have been explained as a functional response to the specific pressures of the habitat: high disturbance, low productivity and relatively strong external subsidies from adjacent aquatic ecosystems [20], [46], [66]. With high levels of rarity, the assemblages represent a valuable component of floodplain biodiversity, and as consumers of emerging invertebrates, a major vector for transporting aquatically derived nutrients into the floodplain. This study has explored some of the complexities inherent in these assemblages, for instance, why dispersal ability and proclivity varies so much between specialist floodplain invertebrates. Variation in feeding strategies and uptake efficiency in an apparently homogenous grouping, extends laterally and longitudinally, partitioning habitat and prey resources. The complexity of floodplain invertebrate communities has been well described, but we are now able to suggest how that complexity translates into important invertebrate functional roles within the floodplain. With an increasing interest in reconnecting floodplains and rivers [67], these invertebrates represent a key functional element in ensuring that such reconnections have demonstrable ecological value.

## Materials and Methods

### Ethics Statement

The landowners gave permission for access to the sites. Permits were not required specifically for the collection of invertebrates at the survey sites. The sampling was based around hand searching thus was of a relatively low intensity and unlikely to have impacts on local populations.

### Study System

**Figure 9:**
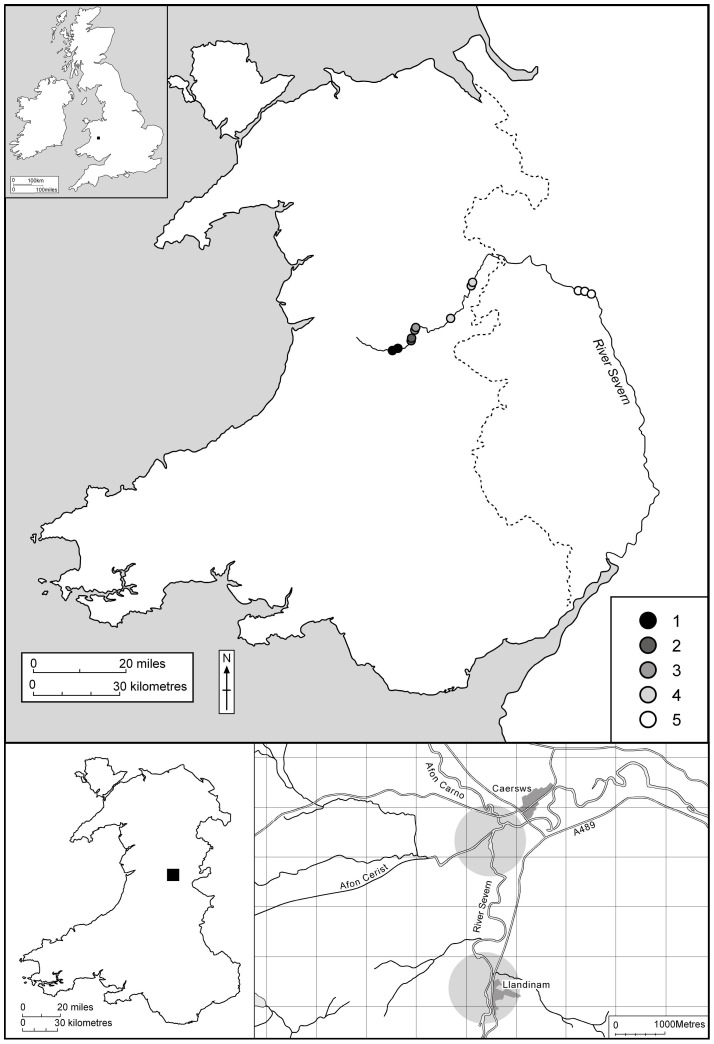
Sample sites on the River Severn, UK, indicating headwater study area containing 20 bars used for inundation data, and the five reaches sampled (15 sites in total) for longitudinal data.

The sampling was nested to include: (i) a detailed study of 20 sampling points on a 5 km stretch of the upper River Severn in mid-Wales (52.5°N, −3.4°E), which contains extensive areas of gravel and sand bars, and (ii) 15 further sampling points along a 150 km stretch of the River Severn, incorporating similar habitat, from the headwaters at Llandiloes, down to Ironbridge Gorge in the English Midlands (Figure 9). Care was taken to avoid sampling bars where livestock had access due to the potential for nutrient enrichment and invertebrate community alteration [68].

Despite impoundment further upstream, the river flow regime retains high variability, sustaining the river's wandering gravel bed (sensu [69]) form within its floodplain, this ensures a high turnover of riparian habitat, utilised by characteristic specialist arthropods. The stretch of the river immediately downstream of Llandinam has been studied extensively for over a decade and is known to contain a diverse and abundant assemblage of specialist invertebrates [20], [44], [70] including dominant ground beetle species (*B. atrocaeruleum*, *B. punctulatum* and *B. tetracolum)* which persist along the 150 km gradient.

### Environmental Variables

A suite of environmental variables were measured on each of the 35 patches (gravel bars). Incline (1–gentle; 2–moderate; 3–steep), area (m^2^), length of wetted edge (m) were measured in situ. Habitat heterogeneity (1–low; 2–moderate; 3–high), vegetation structure (1- bare; 2 – annual/biannual; 3- perennial) and substrate calibre/size measured in Phi classes (1 – coarse gravel; 2 - very coarse gravel) were derived from previous survey data [71]. Inundation susceptibility was assessed by surveying each bar during a period of low flow (April 2009) using a Leica Geosystems 1200 d-GPS for 20 gravel bars in the upper reach of the river. The surveying was done by first walking the outline of each bar, then collecting point data using a 5×5 metre grid, and finally targeting all breaks in slope [72]. These surveys were used to produce a digital elevation model (DEM) of each habitat patch in a GIS (ArcGIS 9.2, ESRI Redlands, USA). Detailed contour maps were produced using splining within ArcGIS Spatial Analyst at 20 cm resolution. The GIS layer was tilted to replicate the water slope through the river reach [38] and related to stage data (river depth) provided by a permanently installed pressure transducer, which recorded data at fifteen minutes intervals throughout the study period (April 2009–April 2010). When compared against available long-term data, the study year shows a comparable hydrograph with peak flows in November-December 2009, lowest flows in April and June, with stochastic high flows events in June, and then August. The GIS and flow data were used to model the area and percentage of habitat submerged under differing river depths, allowing each patch to be assigned an inundation susceptibility value of low (<50% loss of habitat), moderate (51–90% loss) or high (>90% loss) at a river depth 1 m above the April 2009 flow (Figure 2). The validity of the inundation maps was ground-truthed by direct observation across the range of flow events during the sampling period. Pressure transducer data for the site, which indicates local hydrological stage, was examined to assess the speed with which river levels rose. The rising limb of high flow events was consistently between 3.5–5 cm per hour, regardless of timing, magnitude or duration of the inundation event. Given that this rate of increase would take 2–4 hours to submerge even the shallowest profile habitat it is likely that repeated inundation over time would be more important at patch level than single high flow events.

### Invertebrate Sampling and Trait Groups

Samples of numerically dominant terrestrial Coleoptera (Carabidae, Staphylinidae and Coccinelidae) and their potential prey (aquatic and terrestrial) were collected three times during the study (June 2009, September 2009 and April 2010). Terrestrial Coleoptera were collected by hand searching twice at the stream edge, and at the point where perennial vegetation became established on each bar (four searches per bar). Potential terrestrial prey (Collembola,aphids, sedentary Coleopteran larvae (*G. viridula*) and mites, usually parasitic on resident Coleoptera and Aranea) were collected systematically by timed hand searching from the substrate and host plants, taking 10–30 mins per location proportional to the size of the habitat (Table S1). Potential aquatic prey were collected using a standard three minute kick sample with a 500 µm net [73], repeated three times at four positions per season within the sample reaches to incorporate major channel forms (e.g. pools, riffles, glides). All major families of invertebrates were sorted from the samples, with late instar individuals selected for analysis, as they are isotopically closest to adults [46]. Individuals represented Diptera (including Chironomidae, Simuliidae and Tipulidae), Ephemeroptera, Plecoptera and Trichoptera. Although the diversity of potential prey items was reduced in this system, in comparison to studies conducted in European systems, we collected and analysed all potentially important and dominant food sources. For the SIA analyses, orders were separated into families to account for different feeding strategies (e.g. predator/herbivore). As with other published isotope studies, we inferred the signal of a wholly terrestrial-sourced diet from the values derived from predatory beetles with no affinity to the habitat, found away from the stream edge [52]. These possessed a reduced δ^15^N signal relative to gravel bar associated species and the majority of aquatic organisms analysed (Table S1).

In an adaptation of the methodology used by Ribera et al. [56] six specimens of each of twelve dominant sampled Coleoptera had wing, leg and body measurements taken, these were then Ln transformed to attain statistical normality, which was accepted following visualisation and assessment of linearity via QQ plots [74]. These morphological data provided ratios of wing: body and leg: body which were analysed using ANOVA with a post-hoc Tukey test to identify statistically-significantly/similar different groups. Species were grouped according to morphological similarity. To examine the ecological validity of these groupings data from a larger regional study [75] were used to derive Spearman's rank coefficients of species' co-existence based on presence and abundance and significant correlations grouped [76]. Regional variations in assemblage were modelled using generalised linear modelling [77] to further explain longitudinal changes in species' distribution, after assessing normality (via QQ plots) and visual assessment of the presence and importance of outlying data and heterogeneity of variance in graphical outputs from the regression models [74]. This process suggested groupings (Table 1), based on measured traits, modelled distributions and known behaviour [34], [44], [63], and identified target *Bembidion* species for SIA analysis.

### Stable Isotope Analysis

After collection the samples were returned to the laboratory and frozen, prior to identification to species (for Coleoptera) and family (for potential prey) levels. All samples had gut contents removed, were rinsed and dried. Individual samples were split, with one half undergoing lipid extraction prior to analysis for δ^13^C and the other retained for δ^15^N. Lipid extraction was chosen over post-analytical correction methods to reduce the strength of between sample and season variability [78]. A 2∶1 mix of ethanol: methanol was added to samples for a minimum of 30 minutes before centrifuging and disposal of the solvent. This process was repeated three times before the remaining sample was dried for 24 hours at 60°C [79]. Individual samples were then weighed (Carbon: 0.2 mg±0.05 mg: Nitrogen: 0.6 mg±0.06 mg) into tin cups prior to combustion. Stable isotope composition was measured by continuous flow mass spectrometry at the SILLA Laboratory, University of Birmingham using an Isoprime™ IRMS connected to an Elementar PYRO cube©. Precision was ensured by reference to calibrated standards CH3 and N1 from International Atomic Energy Agency (IAEA). The two techniques were analysed on separate sub-samples avoiding observed influences of the lipid extraction process on δ^15^N [80] and precision was better that 0.7‰. The ratios of ^13^C/^12^C and ^15^N/^14^N are presented as relative difference per mil (‰) using the equation:
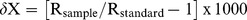
where X = ^13^C or ^15^N, and R = ^13^C/^12^C or ^15^N/^14^N. ^13^C∶^12^C is expressed relative to PDB (Pee Dee Belemnite), where R_standard_ = 1.1237 atom % 13C [81]. ^15^N∶^14^N is expressed relative to atmospheric N_2_, where R_standard_ = 0.3663 atom % ^15^N [82].

### Data Analysis

Species data were analysed separately and by the functional groupings shown in Table 1. Sample sizes were large enough to allow species-specific analysis of three ground beetles with an affinity to the habitat, *B. atrocaeruleum*, *B. punctulatum* and *B. tetracolum*. This study did not attempt to characterise responses of phytophagous specialist species present in the habitat and which sit in the same morphological groupings as predatory *Stenus* spp and *C. 5-punctata*, e.g. *Zorochros minimus* (Boisduval and Lacordaire, 1835) or *Fleutiauxellus maritimus* (Curtis, 1840). Neither did we seek to analyse the fossorial Staphylinids, e.g. *Hydrosmecta* spp. associated with the habitat, due to their small size. These remain areas for potential further exploration but were beyond the scope of the current study.

Analyses were conducted to determine how dietary composition was influenced by habitat variables: inundation susceptibility (Inundation), sampling position (wetted edge or vegetated inland), patch area (Area), sediment calibre (Phi), gradient (incline), vegetation type (Vegetation), wetted perimeter length (Edge) and patch heterogeneity (Heterogeneity), season and longitudinal position along the catchment. The inundation analysis excluded specialist non-ground beetles (group 4) and generalist ground beetles (group 5) due to the small sample numbers retrieved from highly inundated(>90%) patches. This analysis was conducted only on samples collected in autumn 2009, as these represented individuals exposed to known inundation pressures. Correlation between environmental variables and inundation susceptibility was assessed using a Spearman's rank coefficients (Table 3). Where significant correlations occurred, these were assessed for ecological relevance (i.e. which was the stronger driver in the relationship) and individually were run in SIAR to determine their influence upon consumer isotopic signals.

### Isotope Analyses

SIA provides a mechanism for assessing variation in dietary composition both spatially and within assemblages. δ^13^C and δ^15^N are naturally occurring isotopic forms which are fractionated by all organisms during metabolism and excretion [83] allowing for studies of trophic positioning within food webs [84], [85]. Stable Isotope analysis was conducted using a Bayesian mixing model, SIAR (version 4), available as an open source package [86] within R (v 12.3.1) [87]. Isotopic position was assigned using a Bayesian probability framework to evaluate most likely distributions of isotopic values by functional group, data were plotted to provide a visual estimation of trophic positioning via isotopic niche [88]. A refinement of the ‘total area’ concept was used to assess the spatial extent of a food web [84]. Dietary proportions were determined in SIAR in a model fitted via a Markov Chain Monte Carlo (MCMC) method, which provides probability density function distributions of the feasible (total range) and most probable (median) proportions of the organisms' diet. The model captures errors associated with input variables including trophic enrichment factors and source variability, as well as an overall residual error term [86]. As it is not currently feasible to use a multivariate approach, the importance of the environmental variables was examined by adding them individually into the mixing models one variable at a time. The variable that showed the strongest patterns in relation to isotopic values was inundation.

We utilised data from previous gut content and isotopic studies [45], [46], [89] to inform *a priori* selection of potential prey items before repeated modelling produced a final two-source model of aquatic and terrestrial energy sources to riparian invertebrate production. This method reduced the original multisource data set (mean isotopic values of a representative range of these is presented in Table S1), and allowed repeated testing against variables to circumvent the lack of a multivariate component in the mixing model. Trophic enrichment occurs in all consumers, although rates vary between organisms, individuals and tissues [78], [90], [91]. For invertebrates a standard trophic enrichment rate has been established at 2.3‰±0.15 for δ15N and 0.5‰±0.13 δ13C [92], which we included in the mixing models.

## Supporting Information

Table S1 **Mean isotopic values from selection of potential prey items and consumers from both aquatic and terrestrial systems; ranked according to δ^15^N value.**
(DOCX)

## References

[1] Bates AJ, Sadler JP, Henshall S, Hannah DM (2009) Ecology and conservation of arthropods of exposed riverine sediments (ERS). Terrestrial Arthropod Reviews 2: 77–98.

[2] Poff NL, Allan JD, Bain MB, Karr JR, Prestegaard KL, et al (1997) The natural flow regime. Bioscience 47: 769–784.

[3] Gurnell A, Surian N, Zanoni L (2009) Multi-thread river channels: A perspective on changing European alpine river systems. Aquatic Sciences 71: 253–265.

[4] Ward JV, Tockner K, Arscott DB, Claret C (2002) Riverine landscape diversity. Freshwater Biology 47: 517–539.

[5] Nakano S, Murakami M (2001) Reciprocal subsidies: Dynamic interdependence between terrestrial and aquatic food webs. Proceedings of the National Academy of Sciences of the United States of America 98: 166–170. 11136253 10.1073/pnas.98.1.166 PMC14562

[6] Baxter CV, Fausch KD, Saunders WC (2005) Tangled webs: reciprocal flows of invertebrate prey link streams and riparian zones. Freshwater Biology 50: 201–220.

[7] Jardine TD, Kidd KA, Polhemus JT, Cunjak RA (2008) An elemental and stable isotope assessment of water strider feeding ecology and lipid dynamics: synthesis of laboratory and field studies. Freshwater Biology 53: 2192–2205.

[8] Richardson JS, Zhang YX, Marczak LB (2010) Resource Subsidies across the Land-Freshwater Interface and Responses in Recipient Communities. River Research and Applications 26: 55–66.

[9] Tockner K, Stanford JA (2002) Riverine flood plains: present state and future trends. Environmental Conservation 29: 308–330.

[10] Easterling DR, Meehl GA, Parmesan C, Changnon SA, Karl TR, et al (2000) Climate extremes: Observations, modeling, and impacts. Science 289: 2068–2074. 11000103 10.1126/science.289.5487.2068

[11] Tockner K, Paetzold A, Karaus U, Claret C, Zettel J (2006) Ecology of Braided Rivers. In: Smith GS, Best J, Bristow C, Petts G, editors. Braided Rivers: Process, Deposits, Ecology and Management. Oxford: Blackwell Publishing.

[12] Klaar MJ, Maddock I, Milner AM (2009) The development of hydraulic and geomorphic complexity in recently formed streams in Glacier Bay National Park, Alaska. River Research and Applications 25: 1331–1338.

[13] Burt TP, Pinay G (2005) Linking hydrology and biogeochemistry in complex landscapes. Progress in Physical Geography 29: 297–316.

[14] van der Nat D, Schmidt AP, Tockner K, Edwards PJ, Ward JV (2002) Inundation dynamics in braided floodplains: Tagliamento River, Northeast Italy. Ecosystems 5: 636–647.

[15] van der Nat D, Tockner K, Edwards PJ, Ward JV, Gurnell AM (2003) Habitat change in braided flood plains (Tagliamento, NE-Italy). Freshwater Biology 48: 1799–1812.

[16] McCluney KE, Sabo JL (2012) River drying lowers the diversity and alters the composition of an assemblage of desert riparian arthropods. Freshwater Biology 57: 91–103.

[17] Eyre M, Luff M, Lott D (2002) The importance of exposed riverine sediments for phytophagous beetles (Coleoptera) in Scotland and northern England. Aquatic Conservation: Marine and Freshwater Ecosystems 12: 553–556.

[18] Lambeets K, Hendrickx F, Vanacker S, Van Looy K, Maelfait JP, et al (2008) Assemblage structure and conservation value of spiders and carabid beetles from restored lowland river banks. Biodiversity and Conservation 17: 3133–3148.

[19] Sadler J, Bates A (2008) The ecohydrology of invertebrates associated with exposed riverine sediments. In: Wood P, Hannah D, Sadler J, editors. Hydroecology and Ecohydrology: Past, Present and Future: John Wiley & Sons Ltd. pp. 37–56.

[20] Sadler JP, Bell D, Fowles A (2004) The hydroecological controls and conservation value of beetles on exposed riverine sediments in England and Wales. Biological Conservation 118: 41–56.

[21] Anderson J, Hanssen O (2005) Riparian beetles, a unique but vulnerable element in the fauna of Fennoscandia. Biodiversity and Conservation 14: 3497–3524.

[22] Lambeets K, Vandegehuchte ML, Maelfait JP, Bonte D (2008) Understanding the impact of flooding on trait-displacements and shifts in assemblage structure of predatory arthropods on river banks. Journal of Animal Ecology 77: 1162–1174. 18637973 10.1111/j.1365-2656.2008.01443.x

[23] Lambeets K, Vandegehuchte ML, Maelfait JP, Bonte D (2009) Integrating environmental conditions and functional life-history traits for riparian arthropod conservation planning. Biological Conservation 142: 625–637.

[24] Mitsch WJ, Gosselink JG (2000) The value of wetlands: the importance of scale and landscape setting. Ecological Economics 35: 25–33.

[25] Adis J, Junk WJ (2002) Terrestrial invertebrates inhabiting lowland river floodplains of Central Amazonia and Central Europe: a review. Freshwater Biology 47: 711–731.

[26] Thiele H-U (1977) Carabid Beetles in their Environments. Berlin: Springer-Verlag.

[27] Lytle DA, Bogan MT, Finn DS (2008) Evolution of aquatic insect behaviours across a gradient of disturbance predictability. Proceedings of the Royal Society B-Biological Sciences 275: 453–462. 10.1098/rspb.2007.1157 PMC2596821 18055392

[28] Parmesan C, Root TL, Willig MR (2000) Impacts of extreme weather and climate on terrestrial biota. Bulletin of the American Meteorological Society 81: 443–450.

[29] Cornwell WK, Schwilk DW, Ackerly DD (2006) A trait-based test for habitat filtering: Convex hull volume. Ecology 87: 1465–1471. 16869422 10.1890/0012-9658(2006)87[1465:attfhf]2.0.co;2

[30] Desender K (1989) Ecomorphological adaptations of riparian carabid beetles; L'Institut Royal des Science Naturelles de Belgique, Brussels. pp. 309–314.

[31] Andersen J (1985) Low thigmo-kinesis, a key mechanism in habitat selection by riparian Bembidion (Carabidae) species. Oikos 44: 499–505.

[32] Hammond PM (1998) Riparian and floodplain arthropod assemblages: their characteristics and rapid assessment. In: Bailey RG, Jose PV, Sherwood BR, editors. United Kingdom Floodplains: Westbury Publishing. pp. 237–282.

[33] Andersen J (1988) Resource partitioning and interspecific interactions among riparian Bembidion species (Coleoptera: Carabidae). Entomologia Generalis 13: 47–60.

[34] Bates AJ, Sadler JP, Perry JN, Fowles AP (2007) The microspatial distribution of beetles (Coleoptera) on exposed riverine sediments (ERS). European Journal of Entomology 104: 479–487.

[35] Andersen J (2006) Mechanisms in the shift of a riparian ground beetle (Carabidae) between reproduction and hibernation habitat. Journal of Insect Behaviour 19: 545–558.

[36] Kocarek P (2001) Diurnal activity rhythms and niche differentiation in a carrion beetle assemblage (Coleoptera : Silphidae) in Opava, the Czech Republic. Biological Rhythm Research 32: 431–438.

[37] Lundgren JG, Nichols S, Prischmann DA, Ellsbury MM (2009) Seasonal and diel activity patterns of generalist predators associated with *Diabrotica virgifera* immatures (Coleoptera: Chrysomelidae). Biocontrol Science and Technology 19: 327–333.

[38] Paetzold A, Yoshimura C, Tockner K (2008) Riparian arthropod responses to flow regulation and river channelization. Journal of Applied Ecology 45: 894–903.

[39] Andersen J (1968) The effect of inundation and choice of hibernation sites of Coleoptera living on river banks. Norsk Entomologisk Tidsskrift 15: 115–113.

[40] Lavers D, Prudhomme C, Hannah DM (2010) Large-scale climate, precipitation and British river flows Identifying hydroclimatological connections and dynamics. Journal of Hydrology 395: 242–255.

[41] Gerisch M (2011) Habitat disturbance and hydrological parameters determine the body size and reproductive strategy of alluvial ground beetles. Zookeys: 353–370. 10.3897/zookeys.100.1427 PMC3131025 21738421

[42] Hering D, Gerhard M, Manderbach R, Reich M (2004) Impact of a 100-year flood on vegetation, benthic invertebrates, riparian fauna and large woody debris standing stock in an alpine floodplain. River Research and Applications 20: 445–457.

[43] Greenwood MJ, McIntosh AR (2008) Flooding impacts on responses of a riparian consumer to cross-ecosystem subsidies. Ecology 89: 1489–1496. 18589513 10.1890/07-0749.1

[44] Henshall SE, Sadler JP, Hannah DM, Bates AJ (2011) The role of microhabitat and food availability in determining riparian invertebrate distributions on gravel bars: a habitat manipulation experiment. Ecohydrology 4: 512–519.

[45] Hering D, Plachter H (1997) Riparian ground beetles (Coleoptera, Carabidae) preying on aquatic invertebrates: A feeding strategy in alpine floodplains. Oecologia 111: 261–270. 28308003 10.1007/s004420050234

[46] Paetzold A, Schubert CJ, Tockner K (2005) Aquatic terrestrial linkages along a braided-river: Riparian arthropods feeding on aquatic insects. Ecosystems 8: 748–759.

[47] Briers RA, Cariss HM, Geoghegan R, Gee JHR (2005) The lateral extent of the subsidy from an upland stream to riparian lycosid spiders. Ecography 28: 165–170.

[48] Collier KJ, Bury S, Gibbs M (2002) A stable isotope study of linkages between stream and terrestrial food webs through spider predation. Freshwater Biology 47: 1651–1659.

[49] Bastow JL, Sabo JL, Finlay JC, Power ME (2002) A basal aquatic-terrestrial trophic link in rivers: algal subsidies via shore-dwelling grasshoppers. Oecologia 131: 261–268. 28547694 10.1007/s00442-002-0879-7

[50] Post DM (2002) Using stable isotopes to estimate trophic position: Models, methods, and assumptions. Ecology 83: 703–718.

[51] Cremona F, Planas D, Lucotte M (2010) Influence of functional feeding groups and spatiotemporal variables on the delta N-15 signature of littoral macroinvertebrates. Hydrobiologia 647: 51–61.

[52] Paetzold A, Bernet JF, Tockner K (2006) Consumer-specific responses to riverine subsidy pulses in a riparian arthropod assemblage. Freshwater Biology 51: 1103–1115.

[53] Paetzold A, Tockner K (2005) Effects of riparian arthropod predation on the biomass and abundance of aquatic insect emergence. Journal of the North American Benthological Society 24: 395–402.

[54] Laize CLR, Hannah DM (2010) Modification of climate-river flow associations by basin properties. Journal of Hydrology 389: 186–204.

[55] Parnell A, Inge R, Bearhop S, Jackson AL (2008) SIAR: Stable Isotope Analysis in R.

[56] Ribera I, Doledec S, Downie IS, Foster GN (2001) Effect of land disturbance and stress on species traits of ground beetle assemblages. Ecology 82: 1112–1129.

[57] Hering D (1995) Food and competition for food of ground beetles and ants in a north-alpine floodplain. Archiv fuer Hydrobiologie Supplementband 101: 439–453.

[58] Burdon FJ, Harding JS (2008) The linkage between riparian predators and aquatic insects across a stream-resource spectrum. Freshwater Biology 53: 330–346.

[59] Van Looy K, Vanacker S, Jochems H, De Blust G, Dufrene M (2005) Ground beetle habitat templets and riverbank integrity. River Research and Applications 21: 1133–1146.

[60] Mims MC, Olden JD (2012) Life history theory predicts fish assemblage response to hydrologic regimes. Ecology 93: 35–45. 22486085 10.1890/11-0370.1

[61] Petersen I, Masters Z, Hildrew AG, Ormerod SJ (2004) Dispersal of adult aquatic insects in catchments of differing land use. Journal of Applied Ecology 41: 934–950.

[62] Kato C, Iwata T, Wada E (2004) Prey use by web-building spiders: stable isotope analyses of trophic flow at a forest-stream ecotone. Ecological Research 19: 633–643.

[63] Luff M (2007) The Carabidae (ground beetles) of Britain and Ireland. St Albans: Royal Entomological Society.

[64] Gratton C, Forbes AE (2006) Changes in δ^13^C stable isotopes in multiple tissues of insect predators fed isotopically distinct prey. Oecologia 147: 615–624. 16341886 10.1007/s00442-005-0322-y

[65] Tallamy DW, Pesek JD (1996) Carbon isotopic signatures of elytra reflect larval diet in Luperine rootworms (Coleoptera: Chrysomelidae). Environmental Entomology 25: 1167–1172.

[66] Bonn A, Hagen K, Wohlgemuth-Von Reiche D (2002) The significance of flood regimes for carabid beetle and spider communities in riparian habitats - A comparison of three major rivers in Germany. River Research and Applications 18: 43–64.

[67] Palmer MA, Bernhardt ES, Allan JD, Lake PS, Alexander G, et al (2005) Standards for ecologically successful river restoration. Journal of Applied Ecology 42: 208–217.

[68] Bates AJ, Sadler JP, Fowles AP (2007) Livestock trampling reduces the conservation value of beetle communities on high quality exposed riverine sediments. Biodiversity Conservation 16: 1491–1509.

[69] Church M (1983) Pattern of Instability in a wandering gravel bed channel. International Association of Sedimentology Special Publication: 169–180.

[70] Bates A, Sadler J, Fowles A (2006) Condition-dependent dispersal of a patchily distributed riparian ground beetle in response to disturbance. Oecologia 150: 50–60. 16906428 10.1007/s00442-006-0508-y

[71] Bates AJ, Sadler JP (2005) The ecology and conservation of beetles associated with exposed riverine sediments Bangor: CCW.

[72] Brasington J, Rumbsy BT, McVey RA (2000) Monitoring and modelling morphological change in a braided gravel-bed river using high resolution GPS-based survey. Earth Surface Processes and Landforms 25: 973–990.

[73] Winterbourn MJ (1985) Sampling stream invertebrates. In: Pridmore RD, Cooper AB, editors. Biological Monitoring in Freshwaters. Wellington: Miscellaneous Publication No. 83 Water and Soil Directorate.

[74] Zuur AF, Ieno EN, Elphick CS (2010) A protocol for data exploration to avoid common statistical problems. Methods in Ecology and Evolution 1: 3–14.

[75] O'Callaghan MJ (2011) Controls on the distribution of specialist invertebrates inhabiting exposed riverine sediments in England and Wales: University of Birmingham.

[76] Fowler J, Cohen L, Jarvis P (1998) Practical Statistics for Field Biology. Chichester: Wiley.

[77] McCullagh P, Nelder J (1983) Generalized Linear Models. London: Chapman and Hall.

[78] Post DM, Layman CA, Arrington DA, Takimoto G, Quattrochi J, et al (2007) Getting to the fat of the matter: models, methods and assumptions for dealing with lipids in stable isotope analyses. Oecologia 152: 179–189. 17225157 10.1007/s00442-006-0630-x

[79] Folch J, Lees M, Stanley GHS (1957) A simple method for the isolation and purification of total lipides from animal tissues. Journal of Biological Chemistry 226: 497–509. 13428781

[80] Soreide JE, Tamelander T, Hop H, Hobson KA, Johansen I (2006) Sample preparation effects on stable C and N isotope values: a comparison of methods in Arctic marine food web studies. Marine Ecology-Progress Series 328: 17–28.

[81] Craig H (1957) Isotopic standards for carbon and oxygen and correction factors for mass-spectrometric analysis of carbon dioxide. Geochimica Et Cosmochimica Acta 12: 133–149.

[82] Mariotti A (1983) Atmospheric nitrogen is a reliable standard for natural N-15 abundance measurements. Nature 303: 685–687.

[83] Hood-Nowotny R, Knols BGJ (2007) Stable isotope methods in biological and ecological studies of arthropods. Entomologia Experimentalis et Applicata 124: 3–16.

[84] Layman CA, Arrington DA, Montana CG, Post DM (2007) Can stable isotope ratios provide for community-wide measures of trophic structure? Ecology 88: 42–48. 17489452 10.1890/0012-9658(2007)88[42:csirpf]2.0.co;2

[85] Peterson BJ, Fry B (1987) Stable Isotopes in Ecosystem Studies. Annual Review of Ecology and Systematics 18: 293–320.

[86] Parnell AC, Inger R, Bearhop S, Jackson AL (2010) Source Partitioning Using Stable Isotopes: Coping with Too Much Variation. Plos One 5 Article No. e9672. 10.1371/journal.pone.0009672 PMC2837382 20300637

[87] R Development Core Team (2010) R: A language and environment for statistical computing. R Foundation for Statistical Computing, Vienna, Austria. ISBN3-900051-07-0, Available http://www.R-project.org.

[88] Jackson A, Inger R, Parnell A, Bearhop S (2011) Comparing isotopic niche widths among and within communities: SIBER - Stable Isotope Bayesian Ellipses in R. Journal of Animal Ecology. 80: 595–602. 10.1111/j.1365-2656.2011.01806.x 21401589

[89] Davies M (1953) The contents of the crops of some British Carabid beetles. Entomologist's Monthly Magazine 89: 18–23.

[90] Bennett PM, Hobson KA (2009) Trophic structure of a boreal forest arthropod community revealed by stable isotope (delta C-13, delta N-15) analyses. Entomological Science 12: 17–24.

[91] Vander Zanden MJ, Rasmussen JB (2001) Variation in delta N-15 and delta C-13 trophic fractionation: Implications for aquatic food web studies. Limnology and Oceanography 46: 2061–2066.

[92] McCutchan JH, Lewis WM, Kendall C, McGrath CC (2003) Variation in trophic shift for stable isotope ratios of carbon, nitrogen, and sulfur. Oikos 102: 378–390.

