# Supplementary material for: Responses to River Inundation Pressures Control Prey Selection of Riparian Beetles
Source: PLoS One. 2013 Apr 17;8(4):e61866. doi: 10.1371/journal.pone.0061866 (PMC3629232; doi:10.1371/journal.pone.0061866)
Supplement: Table S1 — Mean isotopic values from selection of potential prey items and consumers from both aquatic and terrestrial systems; ranked according to δ15N value. (DOCX) [file pone.0061866.s001.docx]

| \| **Source** \| **Meanδ^15^N** \| **Mean δ^13^C** \| **Source** \| **Feeding** \| \| --- \| --- \| --- \| --- \| --- \| \| Collembola \| 3.50 \| -25.87 \| ERS \| Detrivore^1^ \| \| *Amara aenea* \| 4.61 \| -27.46 \| Floodplain \| Herbivore^2^ \| \| Aphididiae \| 6.00 \| -26.26 \| ERS \| Herbivore^1^ \| \| *Loricera pilicornis* \| 7.04 \| -26.41 \| Floodplain \| Predator^2^ \| \| *Notiophilus biguttatus* \| 7.22 \| -27.07 \| Floodplain \| Predator^2^ \| \| *Agonum meulleri* \| 7.98 \| -26.43 \| Floodplain \| Predator^2^ \| \| Ephemerellidae \| 8.27 \| -30.06 \| Aquatic \| Collector^3^ \| \| *Poecilus cupreus* \| 8.35 \| -26.63 \| Floodplain \| Predator^2^ \| \| *Pterostichus nigrita* \| 8.68 \| -27.81 \| Floodplain \| Predator^2^ \| \| Trichoptera \| 8.72 \| -25.20 \| Aquatic \| Collector^3^ \| \| Heptagenidae \| 9.01 \| -28.47 \| Aquatic \| Scraper^3^ \| \| *Nebria brevicollis* \| 9.20 \| -25.96 \| Floodplain \| Predator^2^ \| \| *Gastrophysa viridula*(larva) \| 9.73 \| -25.77 \| Floodplain \| Herbivore^1^ \| \| Baetidae \| 9.81 \| -29.37 \| Aquatic \| Scraper^3^ \| \| Hydropsychidae \| 10.04 \| -26.11 \| Aquatic \| Collector^3^ \| \| Simuliidae \| 10.08 \| -26.84 \| Aquatic \| Filter^3^ \| \| *Perileptus aurolatus* \| 10.15 \| -25.80 \| ERS \| Predator^4^ \| \| Chironomidae \| 10.60 \| -24.76 \| Aquatic \| Collector^3^ \| \| *Bembidion atrocaeruleum* \| 10.64 \| -26.14 \| ERS \| Predator^4^ \| \| Rhyacophilidae \| 10.97 \| -27.24 \| Aquatic \| Predator^3^ \| \| Leuctridae \| 11.02 \| -25.96 \| Aquatic \| Shredder^3^ \| \| Tipulidae \| 11.22 \| -23.88 \| Aquatic \| Omnivore^3^ \| \| *Bembidion punctulatum* \| 11.54 \| -25.50 \| ERS \| Predator^4^ \| \| Chloroperlidae \| 11.60 \| -24.04 \| Aquatic \| Predator^3^ \| \| Acarina \| 11.70 \| -24.80 \| ERS \| Parasite^4^ \|   ^1^ Potential floodplain prey  ^2^ non-specialist predatory ground beetles  ^3^ potential aquatic prey  ^4^ ERS predators/parasites |  |  |  |  |  |  |
| --- | --- | --- | --- | --- | --- | --- | --- | --- | --- | --- | --- | --- | --- | --- | --- | --- | --- | --- | --- | --- | --- | --- | --- | --- | --- | --- | --- | --- | --- | --- | --- | --- | --- | --- | --- | --- | --- | --- | --- | --- | --- | --- | --- | --- | --- | --- | --- | --- | --- | --- | --- | --- | --- | --- | --- | --- | --- | --- | --- | --- | --- | --- | --- | --- | --- | --- | --- | --- | --- | --- | --- | --- | --- | --- | --- | --- | --- | --- | --- | --- | --- | --- | --- | --- | --- | --- | --- | --- | --- | --- | --- | --- | --- | --- | --- | --- | --- | --- | --- | --- | --- | --- | --- | --- | --- | --- | --- | --- | --- | --- | --- | --- | --- | --- | --- | --- | --- | --- | --- | --- | --- | --- | --- | --- | --- | --- | --- | --- | --- | --- | --- | --- | --- | --- | --- | --- |
|  |  |  |  |  |  |  |
